# 2527: Citation network towards faculty development inside and outside of CTSAs

**DOI:** 10.1017/cts.2017.87

**Published:** 2018-05-10

**Authors:** Solomon Abiola, Kristen Bush

**Affiliations:** University of Rochester Medical Center, Rochester, NY, USA

## Abstract

OBJECTIVES/SPECIFIC AIMS: (1) Obtain publically available citation data, funding data, and generate multiple networks topologies based on dynamic queries of individual faculty. (2) Determine successful pathways that lead to tenure, and career advancement, in addition to determining the effect of CTSA programs on faculty collaboration. (3) Develop publically available commercial interface for the study of faculty networks METHODS/STUDY POPULATION: For our study we included all available citation and funding data publically available on all CTSA programs (as of 2015) with historical data dating back to 2005. We then included the top 25 collegiate institutions who may not have had a CTSA program (eg, Princeton University). We then developed network topologies for each university network, and explore the evolution of individuals in these networks, and the effects of faculty development—as an example in the University of Rochester network, we singled out the directors of the CTSA program there to understand their level of centrality and overall impact on network development, with key observations being that early publications across varying domains lead to stronger network performance. Although individuals who did not benefit from such development, may have succeeded but if they did were likely to leave the institution for elsewhere. RESULTS/ANTICIPATED RESULTS: A secondary goal of this project is to evaluate the effectiveness of the Clinical & Translational Science Institute (CTSI) since its inception in 2006. The mission of CTSI is to advance the field of translational science and research, to link other departments at URMC and community stakeholders by research collaboration, publication, and goals to improve population health, and provide translational education and training to students, researchers, and physicians. To determine how the induction of CTSI affects collaboration within the URMC network, we examined the role of funding in the CTSI network. This was done around the second successful funding around 2013. In doing so we can see that not only did the funding request affect the network topology, but opened new collaborations which were not present prior to the request. DISCUSSION/SIGNIFICANCE OF IMPACT: We have developed an automated method, which is superior to manual methods necessary for citation generation and funding data analysis of faculty growth in citation networks. This technique is applicable to all institutions, not just those in a CTSA environment, but demonstrates the benefit of cross-collaborative efforts, in the case of the URMC network we can state the following. The key takeaway is for individuals to succeed in the URMC collaborative environment they should create their own network and expand it and eventually rise to prominence. There are 2 pathways to this you can take the Dewhurst approach which is to seek out collaborations among internal peers and scale up. Or you can take the Nedergaard approach which is develop the special network, and gain enough public recognition outside of the network that you are capable of leaving it (Fig. 2d). In either case, collaborations among communities and diverse out-degree networks allow faculty to succeed in their given field. Given the wealth of data which has been curated in this fashion, there are numerous explicit questions that can be asked of the data. One of the unique approaches of this data is that is highly reproducible, which allows various questions to be asked. Future work would try to determine what optimal pathways are in a given network to success, and who are ideal collaborators, and collaborations to avoid. Given this information, custom pathways to career success for individual faculty can be developed, moving beyond purely institutional level co-citation networks, which do little to advance faculty development at scale. In Figs 1*c* and *d*, the network increased by 75% in terms of graph density (0.007) and decreased by 18.8% (16) in terms of diameter. What this suggest in that the interconnectivity of the network grew dramatically, while the ability for new members to integrate into it increased. This also apparent when one examines the modularity of the network down by 3.6% (0.857), this suggest that the network has as many communities but these communities are less isolated that those in the previous funding year, meaning fields are becoming more transdisciplinary in their collaborations. This was the result of the presence of a CTSA program, thus demonstrating the effectiveness of such institutions, however, our analysis also lays the framework for applying this to other institutions which may be considering a CTSA. Or maintaining the success of a given CTSA program, and ultimately determining where faculty should place their efforts and choose which programs to pursue career advancement.

